# Glioblastoma stem cells and Wnt signaling pathway: molecular mechanisms and therapeutic targets

**DOI:** 10.1186/s41016-020-00207-z

**Published:** 2020-08-03

**Authors:** Ruoyu Guan, Xiaoming Zhang, Mian Guo

**Affiliations:** 1 Department of Neurosurgery, The Second Affiliated Hospital of Harbin Medical University, 246 Xuefu Road, Nangang, Harbin, 150086 Heilongjiang Province China; 2 Department of Neurology, The Second Affiliated Hospital of Harbin Medical University, Harbin, 150081 Heilongjiang Province China

## Abstract

Glioblastoma is the most common form of primary brain tumor. Glioblastoma stem cells play an important role in tumor formation by activation of several signaling pathways. Wnt signaling pathway is one such important pathway which helps cellular differentiation to promote tumor formation in the brain. Glioblastoma remains to be a highly destructive type of tumor despite availability of treatment strategies like surgery, chemotherapy, and radiation. Advances in the field of cancer biology have revolutionized therapy by allowing targeting of tumor-specific molecular deregulation. In this review, we discuss about the significance of glioblastoma stem cells in cancer progression through Wnt signaling pathway and highlight the clinical targets being potentially considered for therapy in glioblastoma.

## Background

Glioblastoma also known as glioblastoma multiforme (GBM) is one of the most lethal and commonly identified forms of brain tumor [1]. Around 80% of the GBM tumors are located in the cerebral hemisphere (< 5%) located in the cerebellum, brain stem, and spinal cord [2]. GBM are malignant tumors commonly seen in 40–60-year-old individuals [3]. GBMs are often characterized by presence of glioblastoma stem cells (GSCs) [4], which are known to maintain their stemness through a number of signaling pathways [5, 6]. One of pathways gaining attention in GBM is the Wnt signaling. Wnt deregulation in brain is associated with congenital disorders [7], whereas it promotes malignancy in somatic cells of neuronal origin [8].

Wnt signaling pathway is mediated by Wnt family of 19 secreted glycoproteins (bearing22 or 24 cysteine residues) which are essential for cell proliferation, embryonic development, cell polarity, and tissue homeostasis [9]. There are two Wnt pathways which determine the cell fate. Canonical Wnt signaling pathway is essential for embryonic development [10] and non-canonical Wnt pathway regulates cell movement and tissue polarity [11].

The current review focuses on the importance of Wnt signaling pathway in glioblastoma stem cell progression and explores targeting of Wnt signaling molecules as a potential therapy for glioblastoma.

## Glioblastoma stem cells

Glioblastoma stem cells (GSCs) can differentiate into different cell types [12]. GSCs contribute to tumor growth and therapeutic resistance through multiple genetic and epigenetic modifications [4, 13–15]. Genetic modification in GSCs are observed in the form of promotion of angiogenesis [16], promotion of hypoxia through HIF signaling pathway [17], overexpression of surface marker such as SALL4 to inhibit apoptosis [18], or maintenance of multipotency through STAT3 regulation [19]. On the other hand, epigenetic modifications are also reported to be essential for proliferation and survival of GSCs [20]. Hypermethylation of specific genes are reportedly responsible for maintenance of stemness, GSC survival, faster GSC growth, and tumor promotion [21]. Additionally, several miRNAs are reported to induce invasion, migration, self-renewal, stemness, and proliferation in GSCs [22].

### Glioblastoma stem cells and their resistance to chemotherapy and radiotherapy

Chemotherapy and radiotherapy are commonly used adjuvant therapies in GBM patients who have undergone surgery. Despite treatment advances, GBM is associated with high recurrence and reduced time for patient survival [23]. GSCs are implicated for recurrence due to resistance to therapy, thereby allowing cell renewal and tumor re-initiation, while physically, GSCs are difficult to target because of their presence in the perivascular space [24]. At the molecular level, however, resistance to chemo- and radiotherapy is mediated through an aberrant expression of genes responsible for mismatch/base excision repair [25]. Additionally, the role of overexpressed ABC transporters [26] chromatin remodeling [27] and epigenetic modifications at the transcriptional level have also been postulated [28].

The GSC-associated resistance to therapy in GBM is addressed through a triaged approach: one which physically allows drug targets to effectively reach the cells through change in tumor microenvironment [29–33], second which targets GSCs [34, 35] or makes GSCs more susceptible to therapy [36–41], and third through epigenetic modification in GSCs allowing change in tumor characteristics [42–45] such as reduced sphere formation, induction of cellular apoptosis, and prevention of cellular proliferation.

## Wnt signaling pathway

### Canonical Wnt signaling pathway and non-canonical Wnt signaling pathway

Wnt signaling is known as an important regulator of intercellular interaction, cell fate decision, and migration. Developmental defects are attributed to mutations in the critical components of Wnt signaling whereas aberrant Wnt signaling is associated with cancers. Canonical Wnt components bind to cell surface receptors namely frizzled receptor (FZD) and activate low-density lipoprotein receptor-related protein 5, 6 (LRP5/6) complex. Activation of these receptors leads to destabilization of complex consisting of adenomatous polyposis coli (APC), glycogen synthase kinase-3 (GSK3), and Axin [46]. This stabilizes β-catenin, which then travels to nucleus, complexes with T cell factor/lymphocyte enhancer factor, and activates Wnt target genes namely, c-myc, cyclin-D, and VEGF which are important for embryonic development [47–49].

Wnt non-canonical pathway consists of two different pathways depending on the mediators. Disheveled (DVL)-c-jun N terminal kinase (JNK) pathway/planar cell polarity pathway involved in cellular polarity. Binding of wnt to FZD via DVL, JNK kinase activates the pathway responsible for changes in cytoskeleton [50]. The second non-canonical pathway is Ca^2+^-mediated pathway where binding of wnt protein to FZD receptors activates DVL and phospholipase C (PLC) followed by activation of inositol 1,4,5-triphosphate (IP3). IP3 interacts with Ca^2+^ channels in the endoplasmic reticulum and initiates the release of Ca^2+^ ions followed by activation of protein kinase C and cdc42, CAMKII, TAK1, NLK, and NFAT [51–53].

### Role of Wnt signaling pathway in glioblastoma and development of glioblastoma stem cells

Aberrant Wnt signaling is found in various types of cancers. Activation of Wnt pathway is these tumors may be associated with mutations in sentinel components of the pathway viz, APC, β-catenin, AXIN, WTX, and TCF4. Wnt signaling mutations are most extensively characterized in colorectal cancers. It has been observed that close to 85% of colorectal tumors has a loss-of-function mutation in APC whereas 50% of colorectal tumors which lack an APC mutation have activating mutation in β-catenin. Mutations in Wnt signaling components of colon cancer and medulloblastoma were found to be localized in β-catenin, APC, and AXIN1. Mutations of Wnt components are well characterized in various other cancers including hepatocellular carcinoma. Contrastingly, aberration of main components of Wnt pathway is not of common occurrence in GBM, ovarian, head and neck, breast, and gastric cancers [54, 55]. However, recent reports from a small cohort have reported APC mutations in about 13% of GBM cases with a mutation frequency of close to 14.5% [56]. Another independent study identified a homozygous deletion in a negative regulator of Wnt pathway, namely, FAT atypical cadherin 1 (FAT1) [55]. While the role of FAT 1 in GBMs has been explored previously via the PDCD4 [57] and the HIF-1α pathway [58], the study by Morris et al. [55] was the first to demonstrate the influence of FAT1 deletion on Wnt activation.

A recent study pointed to the role of proline-, glutamic acid-, and leucine-rich protein 1 (PELP1), a co-regulator of several nuclear receptor proteins, activating β-Catenin, thereby activating the Wnt signaling pathway. Overexpression of PELP1 was observed in 100 % of the GBM samples [59].

In addition to the mutations in the main components constituting the Wnt pathway, few studies have also identified the role of epigenetic modifications that regulate Wnt pathway. One such study compared the Gene Expression Omnibus microRNA profiling of GBM versus the normal brain and found that *miR-138-2-3p* and *miR-770-5p* were differentially expressed. These two have not been studied for their role in Wnt signaling aberration in GBMs, but their ability to modulate β-catenin has been ascertained in other cancers such as hepatocellular carcinoma and laryngeal cancers [60].

Long noncoding RNAs (lncRNA) namely HOX transcript antisense RNA (HOTAIR), nuclear enriched abundant transcript 1 (NEAT1), and maternally expressed gene 3 were negatively associated; differentiation antagonizing non-protein coding RNA (DANCR) was found to be positively affecting GBM progression through Wnt activation [61].

As discussed above, GBMs are driven by a subset of stem cells that self-renew and attribute tumor heterogeneity in addition to therapeutic resistance. However, the role of Wnt signaling in GBM stem cells remains poorly elucidated. For instance, A study showed that PLAGL2 contributed to stemness in GBM through activation of components of canonical pathway namely, WNT6, FZD9, and FZD2 [62]. Overexpression of PLAGL2 in GBM has also been confirmed. Another study found that the chromatin state in stem cells of GBM led to widespread activation of genes which were normally repressed. One such gene is the ASCL1, a transcription factor that activates Wnt signaling by repressing the negative regulator DKK1. In vivo studies have confirmed the role of ASCL1 in maintenance and tumorigenicity of GBM stem cells [63]. Another study by Adamo et al. found that GBM stem cells significantly overexpressed receptor-like tyrosine kinase (RYK). RYK overexpression influenced stemness frequency, cell migration, and invasion. RYK is postulated to promote stemness in GBM cells through stabilization of β-catenin [64].

Epigenetic modulation of Wnt pathway in GSCs has also been reported. lncRNA MIR22HG was found to be overexpressed in vitro in glioblastoma stem cell lines and has been functionally shown to be responsible for activation of Wnt signaling, by producing *miR-22-3p* and *miR-22-5p* [65]. Contrarily, expression of *miR-34a* negatively correlates with patient survival in GBM. In vitro studies in GSCs have established the role of *miR-34a* expression in targeted degradation of β-catenin. Although the role of *miR-34a* in GSCs has already been established for notch signaling, its role as a modulator of the Wnt pathway has not been reported earlier [66].

While cursory studies exploring the activation of Wnt pathways in GBM and GSCs are reported, an in-depth study for identification of Wnt activators/deregulators either directly or through other pathways in GSCs remains elusive. The data remains further unexplored on patient-derived GSCs since most of these studies are in vitro models of GSC lines. Nevertheless, identification of these genetic or epigenetic modifications that result in aberrant Wnt expression in GSCs may serve as therapeutic or even as prognostic targets.

### Crosstalk between Wnt and other signaling pathways

Wnt signaling pathway plays an important role in many human cancers. It has been reported that there is continuous activation of β-catenin in tumors though no mutations in the major components of the Wnt pathway or alterations in the signaling pathways. These findings confirm that there are others factors capable of activation of β-catenin and its downstream pathways [67]. Epidermal growth factor pathway is essential for motility, growth, proliferation, and differentiation of cells via tyrosine kinase signaling cascade. Overexpression of epidermal growth factor receptor (EGFR) is reported in many types of cancer. EGFR activation and nuclear translocation of β-catenin involves a kinase signaling cascade that leads to disassociation of β-catenin from α-catenin. The free β-catenin translocates to the nucleus and increases tumor invasion [68]. HGF pathways are also found to crosstalk with Wnt signaling pathway. The receptor for HGF is c-met; binding of HGF to c-met phosphorylates β-catenin and translocates it to the nucleus [69]. Notch pathway is reported to repress Wnt pathway during development and homeostasis by associating with and regulating the transcription of β-catenin [70]. Similarly, activation of Wnt pathway antagonizes notch pathway through disheveled pathway leading to deregulation in cancer [71]. Elevated levels of Wnt signaling components in response to hedgehog pathway abnormalities and GLI1 expression are reported in cancer where elevated expression of GLI1 led to the accumulation of β-catenin in nucleus [72]. Hedgehog signaling pathway is also known to play an important role in overexpressing Wnt [73].

### Clinical applications of Wnt signaling pathway targets in glioblastoma

Conventional multimodal treatment strategies employed for GBM have failed to prevent the recurrence and improve patient survival. While these strategies may have worked with other cancers, their failure in part, in GBM, is attributed to GSCs. The lack of therapeutic approach to prevent GSC renewal and promote tumorigenesis influences disease outcomes. Contrarily, targeting GSCs is challenging for its resemblance to normal neuronal stem/progenitor cells. We have reviewed studies which explore targeting GSCs in GBM and their implication on treatment outcome. An in vivo study by Almiron et al. targeted the hypoxic pathway in GSCs. Mice allografted with *S100β-v-erbB/p53*^*−/−*^ glioma stem-like cells were found to have increased survival and decreased cell renewal when treated with agents brefeldin A and EHT-1864 [74].

KDM2B, JmjC domain histone H3K36me2/me1 demethylase, the chromatin regulator, is highly expressed in GBM and plays a role in GSC survival by modulating β-catenin stability. In vitro studies on patient-derived GBM cells demonstrate that genetic or pharmacological inhibition (GSKJ4) of KDM2B results in a decrease of GSC pool along with increased sensitivity to chemotherapy [75]. Several articles have reviewed the targeting of Wnt signaling in GSCs which may be reviewed [76, 77].

### Wnt pathway alteration in GBM

The existing literature establishes that Wnt plays a significant role in maintaining stemness of normal stem cells thereby allowing repair and regeneration. However, a deregulated Wnt pathway may result in onset of cancer stem cells which eventually results in enlarged tumor mass and proliferation. Despite evidence to the de-regulation of Wnt pathway in GBM-associated GSC, the large number of ligands and receptors for either canonical or non-canonical Wnt pathways or cross talking pathways makes systematic classification difficult. However, from currently reviewed studies, it is evident that direct mutations in components of Wnt signaling pathway (APC, β-catenin, AXIN, and TCF4) are not significantly reported. Alternatively, genetic or epigenetic modulation of regulatory pathways influencing Wnt signaling in GSCs is more routinely reported. With new evidence, initial impressions may be changing and thus the significance of Wnt signaling deregulation in generation of GSC in GBM cannot be disregarded.

## Conclusions

GBM is the most lethal form of gliomas involving GSCs. GSCs undergo differentiation, cell proliferation, and self-renewal and maintain stemness by following many signaling pathways. Of these pathways, Wnt signaling pathway is important for differentiation of GSCs. Aberrant Wnt signaling in GSCs renders them resistant to conventional chemo- and radiotherapy thereby making it necessary to assess the efficacy of alternate treatment strategies. While these strategies are experimental in nature, their medical application is limited. The hindrance is the heterogeneity associated with the plasticity of GSC surface markers making recognition and thus targeting of therapy difficult. Additionally, addressing the challenge of similarity between normal neuronal stem cells and GSCs makes treatment-associated toxicity a plausibility. It is suggested that a combinatorial approach which allows cellular targeting of Wnt signaling such as CAR T-mediated drug delivery may be considered to address the challenge.

## Data Availability

All relevant data are within the manuscript.

## Abbreviations

GBM: Glioblastoma multiforme
GSCs: Glioblastoma stem cells
FZD: Frizzled receptor
LRP5/6: Lipoprotein receptor-related protein 5, 6
APC: Adenomatous polyposis coli
GSK3: Glycogen synthase kinase-3
DVL: Disheveled
JNK: Jun N terminal kinase
PLC: Phospholipase C
IP3: Inositol 1,4,5-triphosphate
FAT1: FAT atypical cadherin 1
PELP1: Leucine-rich protein 1
lncRNA: Long noncoding RNAs
HOTAIR: HOX transcript antisense RNA
NEAT1: Nuclear enriched abundant transcript 1
DANCR: Differentiation antagonizing non-protein coding RNA
RYK: Receptor-like tyrosine kinase
EGFR: Epidermal growth factor receptor
GSKJ4: Genetic or pharmacological inhibition
